# Immune Enhancement Effects and Extraction Optimization of Polysaccharides from *Peristrophe roxburghiana*

**DOI:** 10.3390/antiox14091072

**Published:** 2025-09-01

**Authors:** Yong Chen, Zilong Zhao, Yanyan Xu, Fuyan Li, Qiping Zhan

**Affiliations:** 1 College of Agriculture and Biology, Guangxi Minzu Normal University, Chongzuo 532200, China; chenyong@gxnun.edu.cn (Y.C.);; 2 College of Chemical Engineering, Northwest University, Xi’an 710069, China; 3 College of Food Science and Technology, Nanjing Agricultural University, Nanjing 210095, China

**Keywords:** antioxidant, Box–Behnken design, immune enhancing, *Peristrophe roxburghiana*, polysaccharides

## Abstract

The present study aims to optimize the extraction process and systematically investigate the bioactivity of polysaccharides derived from *Peristrophe roxburghiana* (Schult.) Brem. (CPPRs). To this end, the Box–Behnken design–response surface methodology was employed to optimize the extraction parameters of polysaccharides. The optimal extraction conditions were as follows: extraction temperature, 84 °C; extraction duration, 208 min; liquid-to-material ratio, 1:27 g/mL; extraction times, 4 times. The maximum extraction yield reached 17.89%, and the yield under non-optimal extraction conditions is 11–16%. This study systematically investigated the polysaccharides’ physicochemical, structural, and morphological properties using multiple advanced techniques (FTIR, SEM, XRD, HPLC, rheology, and TGA). CPPRs are primarily composed of arabinose, galactose and glucose as the main monosaccharides, amorphous, and capable of low-viscosity gels at low shear rates. Furthermore, CPPRs displayed notable antioxidant activity in vitro, scavenging ABTS^•+^ and DPPH^•^ and reducing Fe^3+^ (with scavenging/reducing rates exceeding 40% at a concentration of 1 mg/mL). Meanwhile, 3 mg/mL CPPRs reduced oxidative damage of red blood cells induced by AAPH, scavenging more than 50% of ROS, and reducing the hemolysis rate by 94.5%. Additionally, CPPRs significantly promoted secretion of cytokines (including TNF-α, IL-6, and IL-10) and NO in RAW264.7 macrophages in vitro compared with the untreated control group. These findings collectively highlight the potential of CPPRs—possessing both antioxidant and immune-enhancing properties—as promising functional ingredients for application in the food and pharmaceutical industries.

## 1. Introduction

Plant polysaccharides, belonging to a class of important biological macromolecules in plants, usually have the functions of regulating intestinal flora, improving antioxidant capacity, regulating inflammatory response, maintaining cell homeostasis, and enhancing immune function [1,2,3,4,5,6], such as antibacterial polysaccharides from *Rhodiola sachalinensis* [7], antioxidant polysaccharides from *Centipeda minima* [8], and immune-enhanced Korean ginseng berry polysaccharides and polysaccharides of *Rose laevigata Michx* fruit [9,10]. The diverse biological activities and functional properties of polysaccharides confer upon them extensive application prospects in the realm of functional foods [11,12]. Food additives (natural antioxidant) are the main application direction of plant polysaccharides [13,14,15,16], which are widely used in the production of beverages, pastries, dairy products, and other foods by improving the taste, color, and stability, such as polysaccharides from *Ziziphus jujuba* cv. Muzaoresidue [17], polysaccharides from *Amomum longiligulare* [18], and polysaccharides from *Osmunda japonica* [19]. Plant polysaccharides have great potential as natural antioxidants, but their research needs to break through the technical challenges of structural analysis, efficient extraction, and functional application to achieve a leap from basic research to industrial application [20,21,22].

Plant polysaccharides, as natural antioxidants, face technical challenges primarily manifested in two aspects: insufficient efficiency in novel polysaccharide screening and challenges in extraction process optimization [23]. In the screening phase, current research predominantly focuses on conventional medicinal plants like shiitake mushrooms and Lycium, with underutilized exploration of agricultural by-products and pharmaceutical process residues [24,25]. Expanding non-traditional sources is critical to accelerating the discovery of high-activity plant polysaccharides. Furthermore, the structural–functional complexity of polysaccharides—characterized by molecular weight heterogeneity, glycosidic bond diversity, and functional group distribution—poses significant challenges in predicting antioxidant activity [26]. By leveraging the combined use of multi-scale analysis techniques (such as NMR, HPLC, and LC-MS for precise determination of monosaccharide composition and glycosidic bond types and AFM, XRD, and SEM for advanced structure analysis), the discovery of the “structure–activity” relationship can be accelerated. In extraction processes, conventional aqueous extraction and alcohol precipitation methods suffer from low yields, high energy consumption, and structural degradation [27,28]. Integrating green technologies with response surface methodology to optimize parameters enables balanced improvements in activity retention and energy efficiency [29]. The establishment of quality control standards based on antioxidant assays facilitates the transition from laboratory-scale research to industrial-scale applications in pharmaceuticals and functional foods, bridging the gap between fundamental studies and practical implementation.

*P. roxburghiana* (Schult.) Brem. is mainly growing in southern China, belonging to the Acanthaceae family and genus *Peristrophe* [30,31,32]. It is one of the natural edible coloring plants used by ethnic minorities in Yunnan, Guizhou, and Guangxi to make “colorful rice” [33,34]. Modern studies have isolated natural organic products such as flavonoids, pigments, terpenoids, and volatile oils from *P. roxburghiana* [30,31,32]. But potential value-added functional compounds natural products of *P. roxburghiana* have not been fully explored. There have been no relevant reports on the extraction and activity verification of polysaccharides derived from *P. roxburghiana* (CPPRs). The extraction of natural CPPRs can further expand the development and utilization of natural active substances from *P. roxburghiana*. Based on the actual application of *P. roxburghiana* as a traditional Chinese medicine (clearing heat and detoxifying, promoting blood circulation, reducing swelling, etc.), selecting antioxidant and anti-inflammatory activities as a means to verify the potential of CPPRs may be more prominent in terms of its targeted nature and application compatibility.

**Figure 1:**
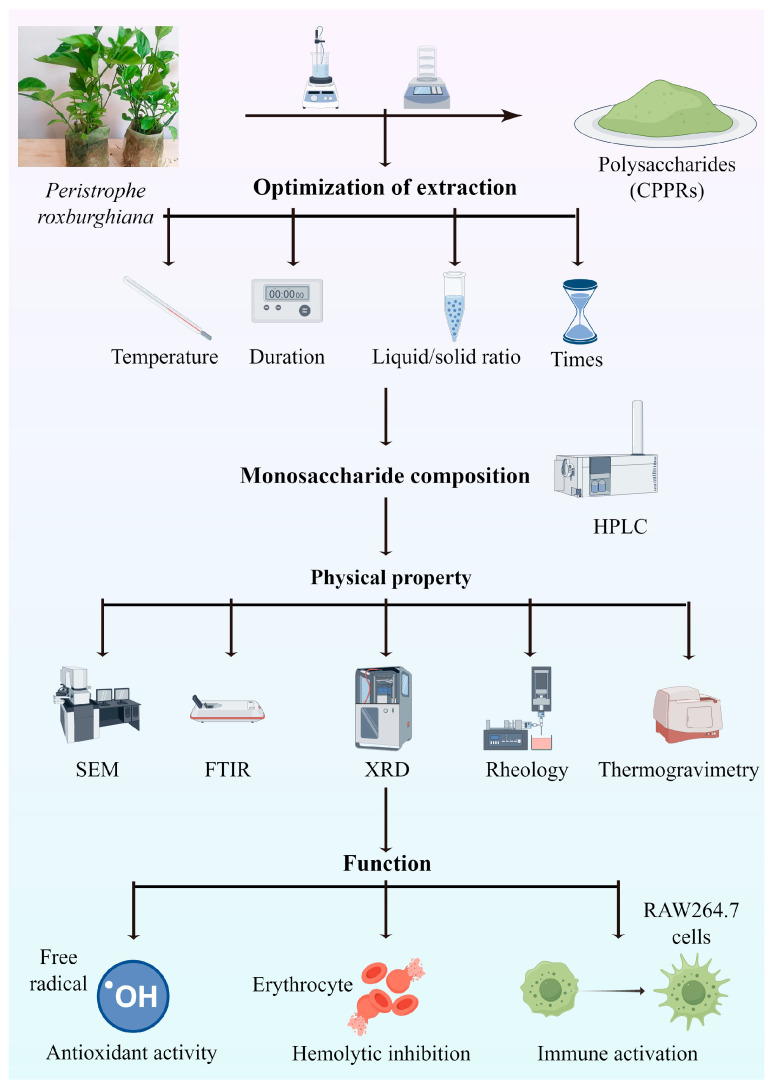
Flow chart of the experiment (by figdraw.com).

The objective of this study was to optimize the extraction conditions—including extraction temperature, extraction duration, liquid-to-material ratio, and extraction times—for highly active polysaccharides derived from the non-traditional medicinal plant *P. roxburghiana* (aimed to serve as potential natural antioxidants), using single-factor tests and Box–Behnken experiments. This study systematically investigated the polysaccharides’ physicochemistry, monosaccharide composition, and morphological properties using multiple advanced techniques: FTIR analyzed chemical bonds and functional groups, SEM visualized microstructures, XRD determined crystal phases, ion chromatography quantified ionic components, rheology evaluated viscoelasticity under shear, and TGA assessed thermal stability. In addition, this study also explored the function (antioxidant and immunopotentiation) of the CPPRs obtained under optimal conditions. These results should provide valuable insights into the extraction yield and physicochemical and functional properties of CPPRs (Figure 1), with potential applications in food industries.

## 2. Materials and Methods

### 2.1. Plant Materials, Chemicals, and Reagents

*P. roxburghiana* was purchased from the Baise herb wholesale market (Guangxi, China). These samples were authenticated by Professor Hui Wu from the South China University of Technology through plant morphology analysis. Guangxi Minzu Normal University’s College of Agriculture and Biology is where voucher specimens (GMN202412) are kept. The fresh leaves were used to extract the polysaccharides required for the experiment. RAW264.7 cells were purchased from Procell Life Science & Technology Co., Ltd. (Wuhan, China). The QuantiCyto^®^ Mouse IL-6 ELISA kit, QuantiCyto^®^ Mouse TNF-α ELISA kit, and QuantiCyto^®^ Mouse IL-10 ELISA kit were purchased from NeoBioscience (Shenzhen, China). The Micro NO Content Assay Kit (BC1475) was purchased from Solarbio (Beijing, China). Lipopolysaccharides (LPS, from *Escherichia coli* 055: B5) and the CCK-8 Cell Proliferation and Cytotoxicity Assay Kit were obtained from Solarbio (Beijing, China). All chemicals used in the extraction process were of chromatography grade to prevent the introduction of reagent contamination.

### 2.2. Extraction of Polysaccharides

The fresh leaves of *P. roxburghiana* were dried at 95 °C, crushed, and passed through a 60-mesh U.S. sieve to obtain fine powder. Hot water was added, and the extract was concentrated to 1/3 of the total by spin distillation. Then, 3 times the volume of anhydrous ethanol was added, followed by alcohol deposition at 4 °C for 12 h. The precipitate was re-dissolved, dialyzed (cut-off molecular weight: 500 Da; time: 48 h; solvent replacement frequency: 12 h/per time), and freeze-dried to obtain CPPRs [35]. The yield of polysaccharides was calculated by using the equation as follows:

yield (%) = (polysaccharide content (g)/weight of dried sample (g)) × 100.

### 2.3. Optimization of CPPR Extraction

To optimize polysaccharide extraction, we used single-factor experiments and Box–Behnken design–response surface methodology (BBD-RSM) [36]. We explored how extraction conditions affected CPPR yield by varying the temperature (50, 60, 70, 80, and 90 °C), duration (60, 120, 180, 240, and 300 min), liquid-to-material ratio (1:10, 1:20, 1:30, 1:40, and 1:50 g/mL), and extraction time (1, 2, 3, 4, and 5 times). Then, based on these results, a three-level–four-factor BBD experiment was performed to fine-tune the extraction parameters [37].

### 2.4. Monosaccharide Composition

The monosaccharide components of CPPRs and monosaccharide standards (Tables S1 and S2; Figures S1 and S2) were analyzed using a Thermo ICS 5000+ ion chromatography system with electrochemical detector. First, 5 mg of polysaccharide sample was weighed, and 1 mL of 2M TFA solution was added. Subsequently, the mixture was hydrolyzed at 121 °C for 2 h, followed by the removal of TFA using nitrogen gas and methanol. A Dionex™ CarboPac™ PA20 (150 × 3.0 mm, 10 μm) liquid chromatography column was employed, with a sample injection volume of 5 μL. The mobile phases consisted of A (H_2_O), B (0.1 M NaOH), and C (0.1 M NaOH with 0.2 M sodium acetate), flowing at a rate of 0.5 mL/min. The column temperature was maintained at 30 °C.

### 2.5. Physicochemical and Thermal Characterization of CPPRs

Purified CPPR powder coated with a thin gold layer was monitored under an SEM (Zeiss Supra 55, Oberkochen, Germany) at an accelerating voltage of 10.0 kV. The image magnification was set as 500×, 1000×, and 5000×. CPPRs were analyzed for the organic functional groups using the potassium bromide pellet method with Fourier-transform infrared spectroscopy (FTIR, Thermo Scientific Nicolet iS10, Waltham, MA, USA) in the 500–4000 cm^−1^ vibration region. X-ray diffraction (XRD, Bruker D8-Advance, Karlsruhe, Germany) was used to determine the products in the range of 5–70° with a rate of 5°/min. Employing the Malvern Kinexus pro+ Rheometer, a 40 mm plate–plate system with a 1 mm gap was used for (1) steady-state flow at 25 °C, with varying shear rates (0.01–1000 s^−1^) to study apparent viscosity vs. shear rate, and (2) dynamic frequency sweep at 25 °C, scanning 0.1–100 rad/s, to assess elastic (G′) and viscous (G″) modulus variations with frequency. The synchronous thermal analyzer (NETZSCH STA 449 F3) was used to perform TG (Thermal Gravimetric) and DSC (Differential Scanning Calorimetry) analyses on CPPRs to characterize their thermal stability, heating them from 30 °C to 910 °C at a rate of 10 °C/min.

### 2.6. Determination of Antioxidant Activity

In vitro antioxidant activity of the samples was determined using ABTS Free Radical Scavenging Capacity Assay Kit (BC4775) and DPPH Free Radical Scavenging Capacity Assay Kit (BC4755, Solarbio, Beijing, China).

Ferric Reducing Power: First, 1 mL of CPPRs (0.2–1.0 mg/mL) was introduced into a test tube, followed by the addition of 2.5 mL of 200 mM phosphate buffer (pH 6.6) and 2.5 mL of 1% potassium ferricyanide. After mixing, the solution was incubated at 50 °C for 20 min, and then 2.5 mL of 10% trichloroacetic acid was added. Centrifugation was conducted at 3000× *g* for 10 min under room temperature. Subsequently, 2.5 mL of the supernatant was combined with 0.5 mL of 0.1% ferric chloride and 2.5 mL of distilled water, mixed for 10 min, and the optical density (OD) value was measured at 700 nm. Distilled water and BHA were, respectively, used as the experimental blank and positive control.

### 2.7. Effect of CPPRs on Erythrocyte Hemolysis Induced by AAPH

Red blood cells (RBCs) were isolated from 10 mL of anticoagulant sheep blood by being centrifuged at 1200× *g* for 10 min at 4 °C. The serum was removed, and the RBCs were washed three times with PBS (pH 7.4). A 20% RBC suspension was prepared using 4x PBS. Samples were dissolved in PBS, and 200 μL of the RBC suspension was mixed with 200 μL of CPPRs (0–3 mg/mL). The mixture was incubated with shaking at 37 °C for 20 min. Then, 400 μL of 200 mM AAPH was added, followed by thorough mixing. Incubation was continued at 37 °C for 2 h. 

After processing, PBS (3.2 mL) was added to the erythrocyte reaction solution of the samples, which were then centrifuged at 1200× *g* for 10 min at 4 °C. The OD value of the supernatant measured at 540 nm was recorded as A. Additionally, 3.8 mL of deionized water was added to 200 μL of a 20% erythrocyte suspension, followed by centrifugation at 1200× *g* for 10 min at 4 °C. The OD value of the resultant supernatant measured at 540 nm was recorded as B. The hemolytic inhibition rate was calculated according to the following formula:

Hemolytic inhibition rate (%) = (1 − A/B) × 100

Erythrocytes were washed 3× with PBS, resuspended (1:5), mixed with DCFH-DA (100 μL: 200 μL, 10 mM), incubated (dark, 37 °C, 25 min), washed 3× again, resuspended in 600 μL PBS, and assayed for fluorescence intensity (FI) (488/525 nm ex/em) to indicate ROS level. PBS-treated erythrocytes served as the control. Relative fluorescence intensity of the sample was measured as (%) = (FI_sample_/FI_control_) × 100.

### 2.8. Cell Culture and Cytotoxicity Assay

RAW264.7 cells were incubated at 37 °C in DMEM with 10% (*v*/*v*) FBS, 100 U·mL^−1^ penicillin, and 100 μg·mL^−1^ streptomycin sulfate under 5% CO_2_. CPPRs’ effects on cell proliferation were tested using the CCK-8 assay. Cells were seeded in a 96-well plate at 1.0 × 10^5^ cells per well, allowed to adhere for 24 h, then treated with CPPRs (50, 100, 200, 400, and 800 μg/mL). After 24 h, CCK-8 solution was added and incubated for 1 h. OD was measured at 450 nm. Control cells were untreated. The relative survival rate of RAW264.7 cells was measured as (%) = ((OD_sample_ − OD_blank_)/(OD_control_ − OD_blank_)) × 100.

### 2.9. Assay of Immune-Enhancement Activity

RAW264.7 cells were seeded into a 96-well plate at 1.0 × 10^5^ cells per well. After 24 h incubation and removing the upper liquid in the orifice plate, CPPRs were added to achieve concentrations of 50, 100, 200, 400, and 800 μg·mL^−1^, respectively. The LPS group (20 ng·mL^−1^) and blank control group were also cultured. After another 24 h, supernatant was collected and measured according to IL-6 ELISA kit, TNF-α ELISA kit, IL-10 ELISA kit, and Micro NO Content Assay Kit.

### 2.10. Statistical Analysis

The data were analyzed by using GraphPad Prism 8.0 and Origin 2017. The response surface design was analyzed using Design Expert 13.0. All experiments were conducted with a minimum of three replicates. The results were expressed as the mean ± standard deviation. Statistical analysis was conducted using one-way analysis of variance (ANOVA) to assess the significance of differences among multiple groups. Duncan’s tests were performed to further compare the statistical means among multiple groups. Different lowercase letters indicated significant differences (*p* < 0.05) among groups in post hoc multiple comparisons.

## 3. Results and Discussion

### 3.1. Extraction and Optimization of CPPRs

#### 3.1.1. Signal-Factor Experimental Analysis

**Figure 2:**
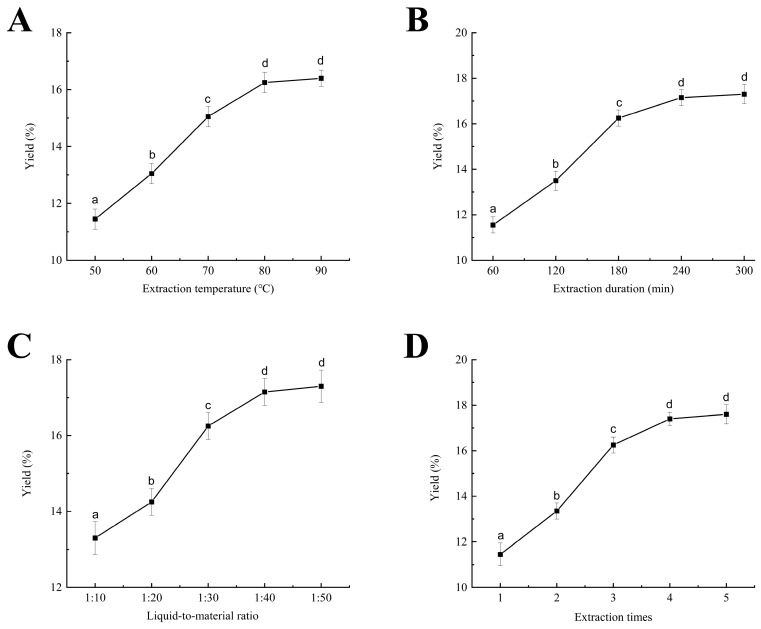
Effect of extraction temperature (**A**), extraction duration (**B**), liquid-to-material ratio (**C**), and extraction times (**D**) on the yield of CPPRs. Data are presented as the mean ± SD. Different lowercase letters indicate significant differences (*p* < 0.05) in multi-range analyses among the groups.

Elevated temperatures compromise plant cell wall integrity and alter cell membrane permeability, thereby facilitating the dissolution of polysaccharides. As the extraction temperature increased from 50 °C to 90 °C, the yield of CPPRs initially increased before reaching a plateau (Figure 2A). At 50 °C (extraction duration of 180 min, liquid-to-material ratio of 1:30, and extraction times of 3 times), the yield was 11.45%, rising to 16.25% at 80 °C, and peaking at 16.40% at 90 °C, with no significant difference (*p* < 0.05). Elevated temperatures may lead to the hydrolysis of certain polysaccharides, thereby limiting the advantages of higher temperatures [38]. Therefore, optimal polysaccharide extraction temperatures for response surface optimization are 75 °C, 80 °C, and 85 °C.

Extraction duration is crucial for optimizing polysaccharide yield, with longer durations leading to more dissolution within a certain range [39]. Initially, the yield rises rapidly, but the rate of increase diminishes over time (Figure 2B). Under conditions (80 °C, 1:30 ratio, 3 times, and 60 min), the CPPR yield was only 11.55%. Extending the extraction time to 180 min increased the yield to 16.25%, while a duration of 240 min resulted in a yield of 17.15%. No significant difference was observed between 180 and 300 min (*p* < 0.05). Once polysaccharides dissolve to a certain level, as further extension will not significantly improve yield, and long durations at high temperatures may decompose them. Consequently, response surface optimization determined that the optimal extraction durations for CPPRs are 150, 180, and 210 min.

The liquid-to-material ratio significantly influences the rate of penetration across cell membranes [8]. Increasing this ratio can enhance the dissolution of CPPRs; however, it also escalates the cost associated with subsequent concentration processes. When the extraction conditions were 80 °C, 180 min, 3 times, and 1:10~1:30, the polysaccharide yield showed a significant increasing trend (*p* > 0.05), from 13.30% at 1:10 to 16.25% at 1:30 (Figure 2C). Nevertheless, further increases in the ratio to 1:40 and 1:50 resulted in only marginal yield improvements to 17.15% and 17.30%, respectively. A higher liquid-to-material ratio enhances the concentration gradient between solute and solvent, thereby accelerating polysaccharide leaching. However, when the solvent volume reaches a certain threshold, the rate of yield increase diminishes. Balancing yield increase with concentration cost, the optimized liquid-to-material ratio was set at 1:25, 1:30, and 1:35.

As the number of extractions increases, the CPPR yield rose; the yield of CPPRs initially rose rapidly and then more gradually (Figure 2D). With fixed conditions (80 °C, 180 min, and 1:30 ratio), the yield increased from 11.45% during the first extraction to 16.25% after three extractions. This increase can be attributed to the re-extraction process, which creates a new concentration gradient of polysaccharides between the interior and exterior of the cell, facilitating the continued dissolution of the remaining polysaccharides [7]. The yields of the fourth and fifth times were 17.40% and 17.60%, respectively, indicating a limited further increase. Hence, response surface analysis focused on two, three, and four extractions.

#### 3.1.2. Response Surface Optimization Experiment Design and Result Analysis

Through single-factor experiment, the optimization level of four factors (including extraction temperature, extraction duration, liquid-to-material ratio, and extraction times) was determined (Table 1). With the yield of polysaccharides as the response value, 29 groups of extraction conditions were designed using Box–Behnken design response surface. The results of each independent variables and polysaccharide yield are shown in Table 2.

**Figure 3:**
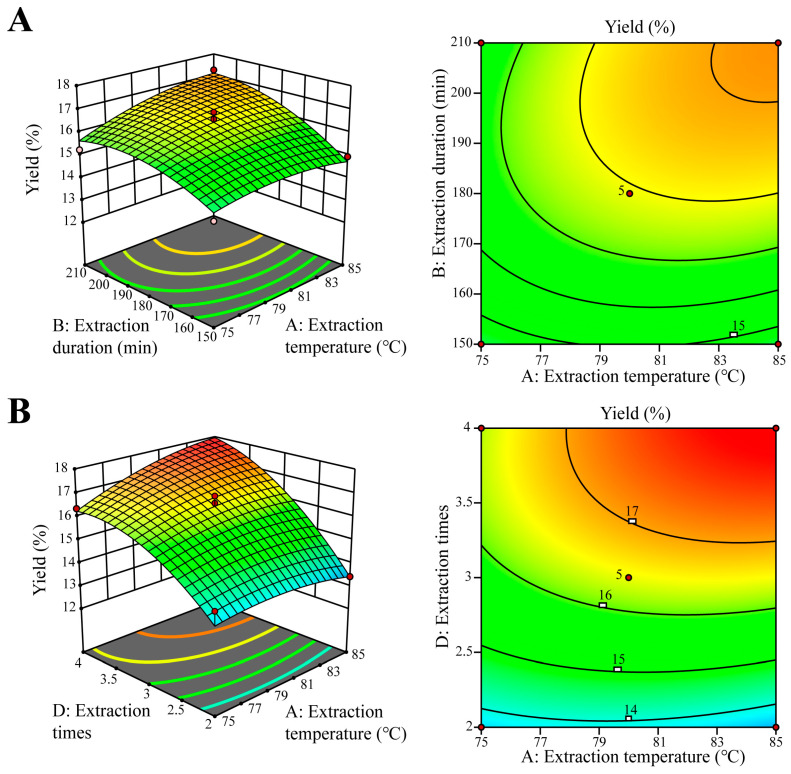
The three-dimensional response surface of the interaction of various factors. (**A**) Three-dimensional response curve graphs and contour graphs for extraction temperature and extraction duration. (**B**) Three-dimensional response curve graphs and contour graphs for extraction temperature and extraction times.

Through regression and fitting, the regression equation was constructed as below: Y = 16.45 + 0.3700A + 0.8242B + 0.7375C + 1.78D + 0.3575AB + 0.0825AC + 0.4850AD + 0.0800BC + 0.1350BD + 0.0600CD − 0.3205A^2^ − 0.5817B^2^ − 0.4443C^2^ − 0.8105D^2^. The analysis results are shown in Table 2. The F-value of 42.33 and the *p*-value (*p* < 0.0001) indicates that the experimental model was significant. The *p* value of 0.4016 and the F-value of 1.39 indicate that the “lack of fit” was not significant relative to the pure error. The R^2^ (R^2^ = 0.9769) and Predicted R^2^ (Predicted R^2^ = 0.8887) indicate that the experimental model has a high fitting degree and high prediction accuracy. The R^2^AdJ = 0.9538 implies that the model has a reasonable agreement with the R^2^ value. The 3D curved surface and response surface contour map visually interpret regression equations, showcasing experimental and interaction levels among independent variables. The curvature’s tilt reflects the impact of two factors on the response, with steeper slopes suggesting stronger interactions. Contour shapes denote interaction strength: ellipses signify significant interaction, whereas circles indicate minimal. According to Figure 3, the contour maps of AB and AD are oval, and the inclinations of their curved surfaces are high, indicating a strong interaction, which is consistent with the analysis of variance results.

From the regression variance (ANOVE) results in Table 3, the model demonstrated was highly significant (*p* < 0.05). According to each *F*-Value, the order of significance of the four factors affecting the response value of polysaccharides was D (extraction times) > B (extraction duration) > C (liquid-to-material ratio) > A (extraction temperature).

Using Design-Expert 13, we obtained the optimal conditions for CPPR extraction: temp 84.45 °C, duration 207.70 min, liquid-to-material ratio 1:27.46, and 3.82 extractions. The predicted max polysaccharide yield was 17.87%. For practicality, we rounded these to 84 °C, 208 min, 1:27, and four extractions. After triplicate experiments, the actual yield was 17.89%, closely matching the theoretical value, validating our optimized parameters.

### 3.2. Monosaccharide Composition of CPPRs

**Figure 4:**
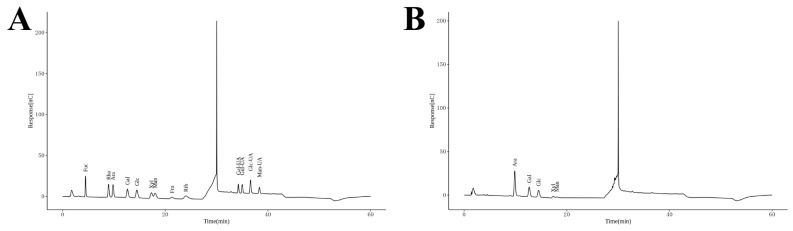
(**A**) Monosaccharide standard and (**B**) monosaccharide composition of CPPRs.

Based on the analysis of peak areas in the chromatogram derived from the mixture of monosaccharides at different gradient mass concentrations and through comparison with the peak areas of polysaccharide samples, the monosaccharide composition of CPPRs was determined to be arabinose (Ara), galactose (Gal), glucose (Glc), xylose (Xyl), and mannose (Man), with molar percentages of 48.92:22.09:17.44:6.85:4.70 (Figure 4 and Table S3). Ara is the dominant monosaccharide in CPPRs. Given Ara’s high stability to heat and acid, CPPRs are likely to exhibit good thermal stability as well. Gal and Glu are present in moderate proportions, typically contributing to the structural stability and biological activity of polysaccharides [40]. Ara, Xyl, and Man share similar activities such as enhancing intestinal health and boosting the immune system, leading to the speculation that CPPRs may also possess functional benefits in these areas.

Based on its monosaccharide composition, the glycosidic linkages of this molecule may exhibit the following structural characteristics: The main chain likely takes Gal linked via β-1,3 or β-1,4 glycosidic bonds as its core structure, resembling the canonical configuration of arabinogalactan [41]. Ara may form branched side chains through β-1,6 glycosidic linkages, connecting to the Gal main chain to generate a highly branched reticular architecture [42]. Glc may copolymerize with the Gal main chain via β-1,4 or α-1,4 glycosidic bonds or act as secondary branching units [43]. Xyl could modify the hydroxyl groups of Gal or Ara through β-1,4 or β-1,3-linked side chains [44]. Man may form terminal branches via α-1,2 or α-1,6 glycosidic linkages or participate in the terminating structures of sugar chains [45]. Collectively, the overall structure may resemble the RG-I domain of pectin or galactomannan, possessing both immunomodulatory and stress-resistant functions.

CPPRs with high proportions of Ara may enhance immune recognition efficiency via mannose receptor (MR) [46]. Gal of CPPRs may activate macrophages through the TLR4/MyD88 pathway, inducing the secretion of pro-inflammatory cytokines. Xyl and Man of CPPRs, on the other hand, augment three-dimensional conformational complexity through their branched structures, promoting multivalent binding to receptors such as Dectin-1 and MR. This facilitates synergistic activation of both TLR4 and CLR pathways, thereby preventing immune imbalance caused by overstimulation of a single receptor. Compared with typical plant polysaccharides (e.g., arabinogalactan or β-glucan), the multi-receptor synergistic activation strategy of CPPRs endows them with dual capabilities: TLR4-dependent pro-inflammatory properties and MR-mediated anti-inflammatory regulatory capacity [47,48].

The monosaccharide composition characteristics of CPPRs make them significantly different from common plant polysaccharides: unlike cellulose and starch, which are structures or energy-storing polysaccharides composed of a single glucose, CPPRs are more likely to belong to a complex and highly branched arabinogalactan. This unique polysaccharide composition indicates that CPPRs have good water solubility and potential prebiotic properties, and are usually closely related to the strong immunomodulatory, antioxidant and other biological activities derived from medicinal plants. Thus, functionally, they have deviated from their traditional roles of providing energy or mechanical support and have become a functional polysaccharide with significant development value.

### 3.3. Analysis of the Physicochemical and Thermal Properties of CPPRs

**Figure 5:**
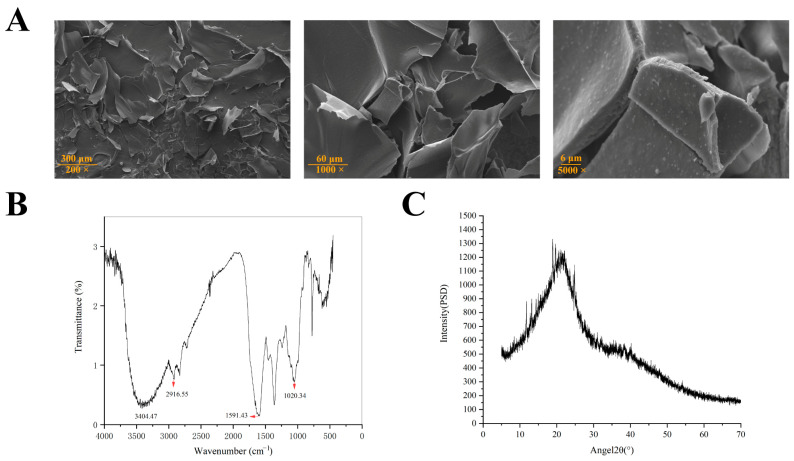
Physicochemical characterization of CPPRs. (**A**) SEM (200×, 1000× and 5000×); (**B**) FTIR; (**C**) XRD.

SEM measurements offer a valuable tool for qualitatively assessing the surface morphology of polysaccharides [49]. The microscopic scanning electron microscope structure of CPPRs was shown in Figure 5A. Predominantly, the polysaccharide exhibits an irregular fragment structure. These fragments possess a rough surface, dense internal structure, and a high degree of adhesion. This observation strongly suggests that CPPRs possess a pronounced amorphous structure yet retain a relatively intact and coherent form.

The FTIR spectrum exhibits polysaccharide-specific bands from 4000 to 500 cm^−1^ [50,51]. Figure 5B displays a strong, broadened absorption peak at 3404.47 cm^−1^, indicating hydroxyl group (O-H) presence, within the range of 3200 to 3650 cm^−1^ for O-H stretching vibration. Additionally, an absorption peak at 2916.55 cm^−1^ suggests the presence of the saturated carbon alkyl group (C-H). The coexistence of these groups suggests the sample possesses the structural features typical of polysaccharides. The absorption peak at 1591.43 cm^−1^ is typically associated with the deformation vibration of sugar rings or the conjugation effect of glycosidic bonds. If the glycosidic bond (such as β- or α-glycosidic bond) forms a conjugated system with the adjacent hydroxyl group, it may cause the stretching vibration frequency of C-O or C-C to decrease to around 1591 cm^−1^. Additionally, the absorption peak near 1020.34 cm^−1^ primarily corresponds to the C-O-C/C-O stretching vibrations of the pyranose ring, serving as a characteristic marker for pyranosidic linkages in polysaccharide structures [52].

X-ray diffraction analysis was used to characterize the structure of the polysaccharide [50]. The CPPRs had a broad dispersion of peaks at a diffraction angle of 20° (Figure 5C). According to the peak shape analysis, they exhibited a lower crystal structure, which was a noncrystalline structure. Due to the length of the molecular chain and the complexity of the interaction between the chains, natural polysaccharides often exhibit the characteristics of a noncrystalline structure. The polysaccharide with a noncrystalline structure may have higher chemical reactivity, be more prone to chemical reaction, and may be more easily enzymolysis or degradation in vivo. Amorphous polysaccharides generally exhibit superior solubility [53]. This enhanced solubility stems from their structural characteristics: lacking long-range ordered crystalline regions, they feature loosely arranged molecular chains and less densely packed hydrogen-bonding networks. These structural attributes render amorphous polysaccharides more prone to interacting with water molecules, facilitating the formation of a stable solvation layer. In industries such as food science and pharmaceuticals, their favorable solubility properties make amorphous polysaccharides a preferred choice as functional additives.

**Figure 6:**
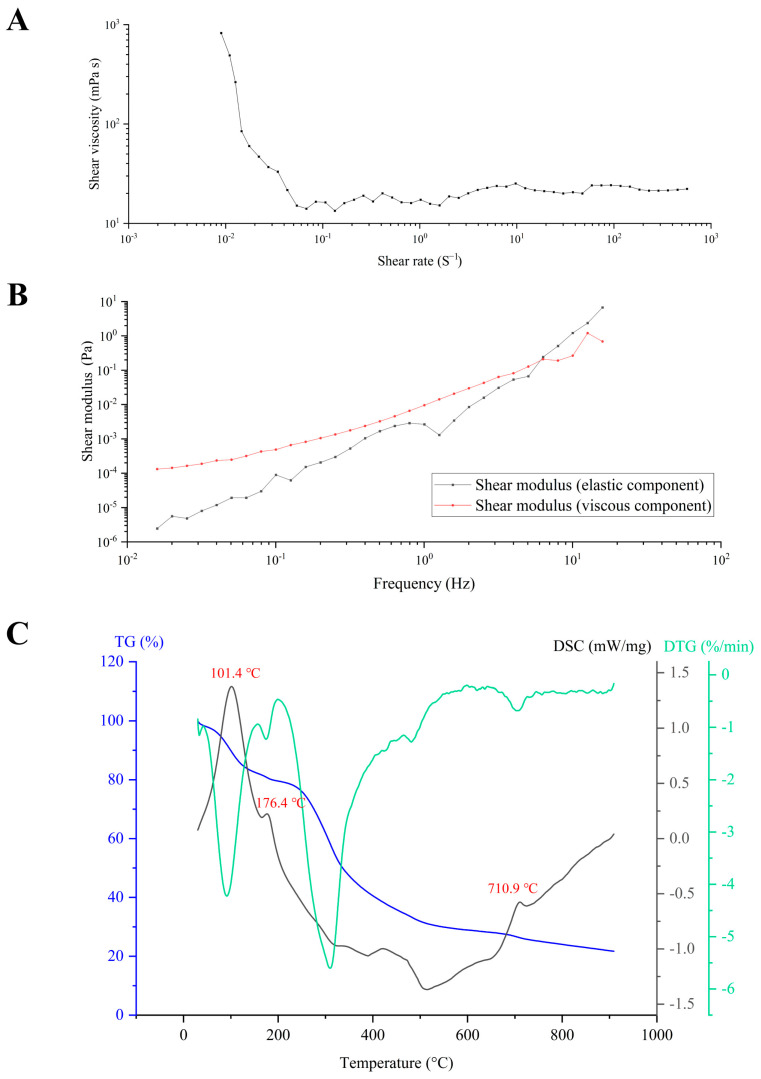
Rheological property and thermal stability of CPPRs. (**A**) Effects of shear rate and concentration on viscosity of CPPRs; (**B**) test results of CPPRs oscillation; (**C**) TG, DTG, and DSC curves of CPPRs.

Polysaccharides, renowned for their exceptional rheological properties, find extensive applications as thickeners, gelling agents, and emulsifiers [13]. Notably, the rheological behavior of polysaccharides is influenced by external factors. Figure 6A investigates the impact of shear rate on the viscosity of CPPRs. At low shear rates, the polysaccharides exhibit typical shear-thinning behavior, where the viscosity decreases with the increase in shear rate, indicative of pseudoplastic fluid characteristics. Conversely, CPPRs under high shear rates are more susceptible to greater shearing forces, yet their viscosity remains virtually unchanged. This suggests that the entangled structure of the long chains in CPPRs undergoes rearrangement under the influence of high shear rates. It may be due to the fact that at low shear rates, the molecular chains form an irregular system through electrostatic resistance, hydrogen bonding, and other interactions, causing their molecular chains to intertwine and generate significant viscous resistance. However, at high shear rates, the random orientation of Brownian motion is disrupted, favoring the directed flow of particles.

Storage modulus, denoted as G′ (Shear modulus (elastic component)), refers to the ability of viscoelastic materials to store energy within a cycle under alternating stress, which is commonly associated with elasticity. In contrast, loss modulus, denoted as G″ (Shear modulus (viscous component)), represents the capacity of dissipating energy within a cycle of variation, typically associated with viscosity [54]. In this experiment, frequency oscillation was employed to test the viscoelastic properties of CPPRs (Figure 6B). Across the entire frequency range of 0.01 to 6 Hz, G′ and G″ of CPPRs increase with frequency, with G′ consistently remaining higher than G″. This indicates that CPPRs exhibit elastic behavior, characteristic of a gel-like nature. At this stage, the CPPR solution is likely to form a gel network, and the stability of this gel is partly dependent on its concentration. The gel-like properties displayed are likely related to its three-dimensional network structure, functional groups, degree of crosslinking, and polymerization. Within the frequency range of 6 to 16 Hz, both G′ and G″ of CPPRs continue to increase with frequency, yet G′ consistently falls below G″. This suggests that during deformation, more energy is dissipated as heat or other forms, indicative of CPPRs displaying stronger liquid-like properties. Consequently, the material exhibits a heightened tendency to dissipate energy, resulting in a more pronounced fluidic behavior.

The thermogravimetric (TG) curve represents the correlation between the sample mass and temperature under a specific heating program, providing a direct visualization of the stages, trends, and magnitudes of mass changes in the sample [55]. Typically, in a DSC (Differential Scanning Calorimetry) curve, convex peaks signify endothermic reactions, while concave peaks indicate exothermic reactions, corresponding to increases or decreases in enthalpy values [56]. The DTG (Derivative Thermogravimetry) curve, on the other hand, is the first-order derivative of the TG curve, reflecting the rate of mass change with respect to temperature and time. As depicted in Figure 6C, the mass and heat changes of CPPRs primarily undergo three distinct stages within the temperature range of 30 °C to 910 °C. The first stage of CPPRs spans from 30 °C to 165 °C, with a temperature inflection point, known as the glass transition temperature (Tg), at 101.4 °C. For polymers, Tg represents the temperature at which the material transitions from a glassy state to a rubbery state, marking the highest temperature before the material softens. This transition corresponds to an endothermic peak on the DSC curve. Evidently, CPPRs can maintain their glassy state at room temperature, indicating thermal stability during storage. According to the TG curve, the mass loss of CPPRs in the first stage is 18.5%, which is mainly due to the loss of bound water. The temperature ranges for the second and third stages of CPPRs are 165–509 °C and 509–694 °C, respectively. During these stages, the primary event is the intense thermal decomposition of polysaccharide molecules, accompanied by the breakage of hydrogen bonds, C-O bonds, and C-C bonds. This process is exothermic. Compared with other similar polysaccharides (such as starch, cellulose, etc., which are common polysaccharides), the Tg (101.4 °C) of CPPRs is usually similar to that of common polysaccharides available on the market (such as the Tg of anhydrous corn starch and pea starch is approximately 95–110 °C). Its thermal decomposition initiation temperature (165 °C) and overall decomposition temperature range (165–694 °C) also exhibit a wider high-temperature stability, demonstrating its superior structural retention ability in high-temperature environments.

### 3.4. Antioxidant Activities of CPPRs

The principle of the ABTS radical scavenging assay is based on the ability of antioxidant compounds to convert radicals into non-radical forms by donating electrons and hydrogen atoms [57,58,59]. It is a widely used method for screening the antioxidant activity of both lipophilic and hydrophilic compounds. The DPPH method is the “gold standard” for antioxidant activity research, especially suitable for rapid screening and preliminary assessment. Its core principle is based on the scavenging ability of DPPH free radicals [59,60]. The Fe^3+^ reducing power, which involves converting the Fe^3+^/ferricyanide complex to its ferrous form by donating an electron or hydrogen atom, is also a common method for evaluating the antioxidant activity of compounds [61,62].

**Figure 7:**
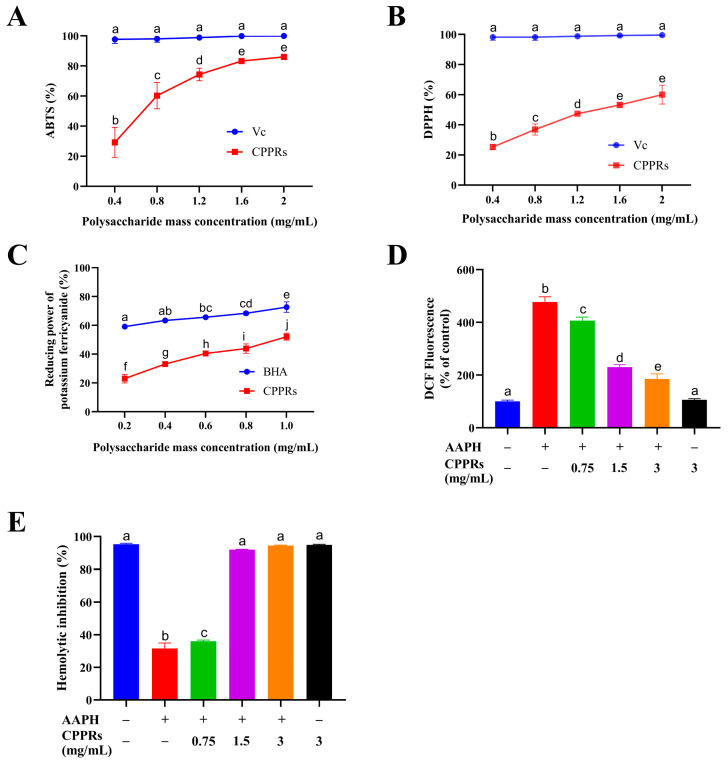
Antioxidant activities of CPPRs. (**A**) ABTS^•+^ radical scavenging capacity; (**B**) DPPH radical scavenging capacity; (**C**) ferric reducing antioxidant power; (**D**) ROS levels of blood cells after treatment; (**E**) hemolytic inhibition rate of CPPRs. Data are presented as the mean ± SD. Different lowercase letters (a, b, c, etc.) indicate significant differences (*p* < 0.05) in multi-range analyses among the groups.

As depicted in Figure 7A,B, the scavenging efficacy of CPPRs against ABTS^•+^ and DPPH radicals exhibits a clear linear correlation with their concentration, increasing as the concentration rises. Within the concentration range of 0.4 to 2 mg/mL, the scavenging rate of CPPRs for the ABTS^•+^ radical increases from 29.8% to 86.03%, although it remains slightly less effective than an equivalent concentration of a Vc solution. Similarly, within the same concentration range, the scavenging rate for the DPPH radical increases from 25.31% to 60.03%, again demonstrating slightly lower potency compared to a Vc solution at equivalent concentrations. This discrepancy may be ascribed to differences in molecular structure and mode of action: as small-molecule compounds, Vc can directly neutralize free radicals by donating hydrogen atoms or electrons; in contrast, CPPRs, likely due to its larger molecular weight or the presence of steric hindrance, demonstrates marginally reduced binding efficiency toward free radicals. Similarly, Figure 7C reveals a direct correlation between the concentration of CPPRs and their reducing ability towards Fe^3+^, with the scavenging rate increasing from 21.5% to 52.05% within a range of 0.2 to 1 mg/mL. These findings underscore the excellent reducing activity possessed by CPPRs.

The clearance effects of CPPRs on ABTS^•+^ and DPPH free radicals, as well as their reducing ability towards Fe^3+^, all exhibit significant concentration-dependent characteristics. This highlights their outstanding reducing activity, but their mechanism of action differs fundamentally from that of small molecule antioxidants such as Vc; this difference is rooted in their unique molecular structure, which is the most fundamental difference compared to other common plant polysaccharides: Unlike starch (100% glucose) as a simple energy polymer or cellulose as a structural polymer, CPPRs have a highly branched arabinogalactan structure centered on arabinose (48.92%) and galactose (22.09%), forming a large number of exposed active hydroxyl groups that can neutralize free radicals through mechanisms such as hydrogen atom transfer. At the same time, due to their large molecular weight and spatial steric hindrance effect, their action efficiency is different from that of small molecule Vc. Furthermore, compared with another common type of polymeric compound—pectin with galacturonic acid as the main chain—the neutral sugar-dominated structure of CPPRs gives it different charge properties and action modes. Compared with many simple or inert common polysaccharides (such as starch, which has almost no activity), the complex and highly branched polymeric conformation of CPPRs provides a significant basis for biological activity, making it not only a dietary fiber but also a natural antioxidant with development potential, which is precisely the key that distinguishes it from most common plant polysaccharides in terms of functional applications.

### 3.5. Analysis of the Effect of CPPRs on Erythrocyte Hemolysis Induced by AAPH

Oxidative stress plays a pivotal role in numerous physiological and pathological phenomena in humans [63]. AAPH, an effective peroxyl radical initiator, can continuously generate peroxyl radicals by controlling its concentration [64]. The AAPH-induced erythrocyte hemolysis model is a reliable cellular model widely used to study oxidative damage to biomembranes. Consequently, this model is employed to investigate the antioxidant activity of CPPRs at the cellular level and their underlying mechanisms of action.

The protective effect of active substances on erythrocyte hemolysis is generally ascribed to their ability to inhibit intracellular ROS production [65]. DCFH-DA was utilized to quantitatively assess the inhibitory effect of various concentrations of CPPRs on AAPH-induced ROS production in erythrocytes. DCFH-DA, a non-fluorescent deriv., converts to DCFH via nonspecific esterases in cytoplasm. AAPH-generated ROS oxidizes DCFH to DCF. Hence, DCF fluorescence intensity correlates positively with ROS levels [66]. Notably, AAPH treatment significantly elevated ROS levels in erythrocytes, which were effectively reduced by CPPRs in a dose-dependent manner (Figure 7D). At a CPPR concentration of 3 mg/mL, the ROS level, measured at 185% relative to the negative control, was significantly diminished compared to the model group, which exhibited ROS levels at 477% of the negative control.

RBCs, which are rich in hemoglobin and possess a large cell membrane surface area, are highly vulnerable to oxidative stress-induced hemolysis, resulting in the release of free hemoglobin into the plasma. AAPH demonstrates a hemolysis inhibition rate of only 31.60% in RBCs. However, as the concentration of CPPRs increases from 0 to 3 mg/mL, the hemolysis inhibition rate significantly exceeds 94.50%. Furthermore, in the absence of AAPH, treatment of RBCs with the highest concentration of CPPRs achieves a hemolysis inhibition rate comparable to that of normal RBCs treated with PBS alone, indicating that CPPRs are non-toxic and do not cause RBC damage (Figure 7E).

### 3.6. Cell Cytotoxicity

**Figure 8:**
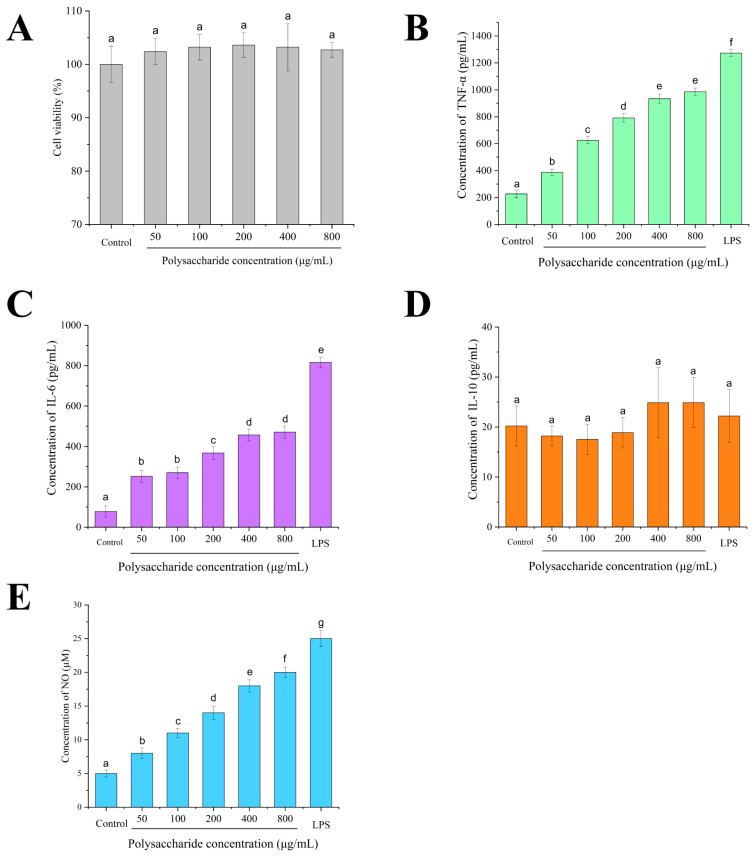
Stimulation effects of CPPRs on the RAW264.7 cells. (**A**) Cytotoxicity of polysaccharides toward RAW264.7 cells for 24 h; effects of CPPRs on the secretion of TNF-α (**B**), IL-6 (**C**), IL-10 (**D**), and NO (**E**) in the RAW264.7 cells. Data are presented as the mean ± SD. Different lowercase letters indicate significant differences (*p* < 0.05) in multi-range analyses among the groups.

The results demonstrated that CPPRs exhibited no significant proliferative effects (*p* > 0.05) on RAW264.7 macrophages across the tested concentration range (50–800 μg/mL) (Figure 8A). Notably, 200 μg/mL CPPRs maintained optimal cell viability (103.6 ± 2.3%), suggesting that their biological functions may primarily reside in immunophenotypic modulation and functional activation rather than direct mitogenic activity. Importantly, no cytotoxic effects were observed at the highest concentration (800 μg/mL), with no significant differences in cell viability compared to other treatment groups.

### 3.7. Effect of CPPRs on Immune-Enhancement Activity in RAW264.7 Macrophages

Plant polysaccharides promote the production of interleukins and interferons, immune factors that play an important role in regulating the immune response [67]. TNF-α, IL-10, and IL-6 are immune cytokines secreted by macrophages, which have great impact on the immune function of the body [68,69]. And NO can induce the production of various cytokines, participate in various physiological and pathological processes of the body, and is also an important indicator of the activation of immune cells [70].

The effect of CPPRs on the secretion of TNF-α, IL-10, IL-6, and NO by RAW264.7 cells is shown in Figure 8B–E. Compared with the control group, in the concentration range of 50–800 μg/mL, the secretion levels of TNF-α, IL-6, and NO were also increased with the increase in the concentration of CPPRs but to a lesser extent than LPS. Especially at 400 μg/mL mass concentration, TNF-α, IL-6, and NO reached 2250 pg/mL, 910 pg/mL, and 42 pM, respectively, indicating that CPPRs could activate RAW264.7 macrophages and promote the production of TNF-α, IL-6, and NO. However, no statistically significant differences were observed in IL-10 secretion levels among RAW264.7 macrophages subjected to various treatments. These findings thus suggest that under the current experimental conditions, the immunomodulatory effects of CPPRs may primarily manifest in other phenotypic indicators—such as phagocytic capacity, NO production, and M1/M2 marker expression ratios—rather than directly promoting IL-10 release.

These findings prompt a critical re-evaluation of CPPRs’ immunomodulatory mechanisms. While conventional plant polysaccharides typically activate pathways such as TLR4/MyD88 to concomitantly induce both pro-inflammatory and anti-inflammatory cytokines, CPPRs’ failure to stimulate IL-10 production suggests their engagement of distinct receptor systems or downstream signaling nodes. Given the concomitant elevation in NO output and its established role in cytokine network regulation, CPPRs may function through iNOS-NO axis activation to drive a TNF-α/IL-6 positive feedback loop while bypassing IL-10-associated regulatory pathways. This selective activation profile aligns precisely with macrophage polarization theory: marked upregulation of pro-inflammatory mediators (TNF-α, IL-6, and NO) coupled with baseline anti-inflammatory factor (IL-10) expression strongly indicates that CPPRs preferentially induce M1-type macrophage activation rather than M2 polarization. Consequently, CPPRs’ immunomodulatory effects likely manifest primarily through non-cytokine-dependent pathways, such as enhanced phagocytic capacity, ROS generation, or altered M1/M2 marker expression ratios. Future studies should employ transcriptomic profiling and signaling pathway inhibitors to specifically investigate whether CPPRs achieve this differential activation by modulating the balance transcriptional regulators.

## 4. Conclusions

This study was the first to conduct extraction optimization, physical characterization, and activity investigation of CPPRs. The optimized extraction of CPPRs yielded 17.89% under response surface-derived conditions. CPPRs displayed notable antioxidant activity, scavenging ABTS^•+^ and DPPH^•^, reducing Fe^3+^, and reducing oxidative damage of red blood cells. The CPPRs also showed an excellent immunoenhancement activity on RAW264.7 cells, promoting the secretion of TNF-α and IL-6 and increasing the content of NO. These results demonstrate the potential applications of CPPRs with antioxidant and immune-enhancing properties in the food and pharmaceutical industries.

Critical limitations of this study include the following: (1) The mechanism of action remains unverified through proteomics or genetic knockdown. (2) No human primary macrophage validation or in vivo dose–response correlation studies were conducted. (3) The metabolic stability during the digestion process has not been determined.

Based on the limitations of relevant research, future CPPR research should focus on four priorities: (1) developing advanced extraction methods like AI-optimized enzymatic hydrolysis, (2) resolving precise structure–activity relationships through HRMS and 2D-NMR analysis of glycosidic patterns, (3) validating in vivo efficacy using aging models with optimized delivery systems to enhance bioavailability, and (4) expanding into functional foods or targeted drug carriers to leverage translational applications. These integrated approaches will accelerate the development of health industry applications.

## Figures and Tables

**Table 1:** Independent variables and their levels used for Box–Behnken central composite design.

	Levels		
	−1	0	1
Extraction temperature (°C) (A)	75	80	85
Extraction duration (min) (B)	150	180	210
Liquid/solid ratio (g/mL) (C)	1:25	1:30	1:35
Extraction times (D)	2	3	4

**Table 2:** Box–Behnken central composite design for independent variables and their response.

Runs	A: Extraction Temperature (°C)	B: Extraction Duration (min)	C: Liquid-to-Material Ratio (g/mL)	D: Extraction Times	Polysaccharide Yield (%)
**1**	0	0	1	−1	13.77
**2**	1	0	0	−1	13.42
**3**	0	0	0	0	16.34
**4**	0	0	1	1	17.86
**5**	1	0	0	1	17.48
**6**	0	1	0	1	17.86
**7**	−1	0	1	0	15.98
**8**	0	0	0	0	16.58
**9**	0	1	−1	0	15.49
**10**	−1	0	−1	0	14.67
**11**	1	0	−1	0	15.28
**12**	0	0	0	0	16.25
**13**	0	1	1	0	17.18
**14**	0	0	0	0	16.88
**15**	0	−1	−1	0	13.93
**16**	1	0	1	0	16.92
**17**	−1	0	0	−1	14.23
**18**	0	0	−1	−1	12.47
**19**	0	0	0	0	16.18
**20**	−1	0	0	1	16.35
**21**	1	1	0	0	17.25
**22**	−1	−1	0	0	14.38
**23**	0	0	−1	1	16.32
**24**	1	−1	0	0	14.95
**25**	0	−1	0	1	15.95
**26**	0	−1	1	0	15.30
**27**	0	−1	0	−1	12.58
**28**	−1	1	0	0	15.25
**29**	0	1	0	−1	13.95

**Table 3:** ANOVA for response surface quadratic model analysis of variance.

Source	Sum of Squares	Sum of Squares	Mean Square	*F*-Value	*p*-Value Prob > *F*	Significant
Model	61.95	14	4.42	42.33	<0.0001	**
A: Extraction temperature	1.64	1	1.64	15.72	0.0014	**
B: Extraction duration	8.15	1	8.15	77.98	<0.0001	**
C: Liquid-solid ratio	6.53	1	6.53	62.44	<0.0001	**
D: Extraction times	38.16	1	38.16	365.09	<0.0001	**
AB	0.5112	1	0.5112	4.89	0.0441	*
AC	0.0272	1	0.0272	0.2605	0.6178	——
AD	0.9409	1	0.9409	9.00	0.0095	**
BC	0.0256	1	0.0256	0.2449	0.6284	——
BD	0.0729	1	0.0729	0.6974	0.4177	——
CD	0.0144	1	0.0144	0.1378	0.7161	——
A^2^	0.6663	1	0.6663	6.37	0.0243	*
B^2^	2.20	1	2.20	21.00	0.0004	**
C^2^	1.28	1	1.28	12.25	0.0035	**
D^2^	4.26	1	4.26	40.76	<0.0001	**
Residual	1.46	14	0.1045	——	——	——
Lack of fit	1.14	10	0.1137	1.33	0.4213	——
Pure error	0.3267	4	0.0817	——	——	——
Cor. total	63.41	28	——	——	——	——

** *p* < 0.01; * *p* < 0.05. “——“ represents the absence of any calculable or meaningful numerical value.

## Data Availability

Data are contained within the article and Supplementary Materials.

## Supplementary Materials

The following supporting information can be downloaded at: https://www.mdpi.com/article/10.3390/antiox14091072/s1, Figure S1. The liquid chromatograms of the monosaccharide standard samples. Figure S2. Standard curves for each monosaccharide (Ara, Gal, Glc, Man, and Xyl). The abscissa represents the mass concentration (μg/ml), and the ordinate represents the peak area of the liquid chromatography (nC*min). Table S1. Information on the standard substances of this project. Table S2. Information on the standard substances of this project. Table S3. Monosaccharide composition of CPPRs. Table S3. Monosaccharide composition of CPPRs.

## Abbreviation

AAPH: 2,2′-azobis [2-methylpropionamidine] dihydrochloride; AFM, atomic force microscope; Ara: arabinose; BHA: 3-tert-butyl-4-hydroxyanisole; CLR: C-type lectin receptor; CPPRs: polysaccharides derived from Peristrophe roxburghiana (Schult.) Brem; DSC, differential scanning calorimetry; FTIR, Fourier-transform infrared spectroscopy; Gal: galactose; Glc: glucose; Man: mannose; MR: mannose receptor; NMR: nuclear magnetic resonance; OD, optical density; ROS, reactive oxygen species; SEM, scanning electron microscope; TFA, trifluoroacetic acid; TG, thermal gravimetric; XRD, X-ray diffraction; Xyl: xylose.

## References

[1] Murphy E.J., Fehrenbach G.W., Abidin I.Z., Buckley C., Montgomery T., Pogue R., Murray P., Major I., Rezoagli E. (2023). Polysaccharides—Naturally Occurring Immune Modulators. Polymers.

[2] Schepetkin I.A., Quinn M.T. (2006). Botanical polysaccharides: Macrophage immunomodulation and therapeutic potential. Int. Immunopharmacol..

[3] Wan X., Yin Y., Zhou C., Hou L., Cui Q., Zhang X., Cai X., Wang Y., Wang L., Tian J. (2022). Polysaccharides derived from Chinese medicinal herbs: A promising choice of vaccine adjuvants. Carbohyd. Polym..

[4] Zhao Y., Yan B., Wang Z., Li M., Zhao W. (2020). Natural Polysaccharides with Immunomodulatory Activities. Mini Rev. Med. Chem..

[5] Yin M., Zhang Y., Li H. (2019). Advances in Research on Immunoregulation of Macrophages by Plant Polysaccharides. Front. Immuol..

[6] Chen X., Zhang J.S., Wang Y.F., Hu Q.H., Zhao R.Q., Zhong L., Zhan Q.P., Zhao L.Y. (2024). Structure and immunostimulatory activity studies on two novel *Flammulina velutipes* polysaccharides: Revealing potential impacts of →6)-α-d-Glcp(1→ on the TLR-4/MyD88/NF-κB pathway. Food Funct..

[7] Hao Y.-J., Zhang K.-X., Jin M.-Y., Piao X.-C., Lian M.-L., Jiang J. (2023). Improving fed-batch culture efficiency of Rhodiola sachalinensis cells and optimizing flash extraction process of polysaccharides from the cultured cells by BBD–RSM. Ind. Crop. Prod..

[8] Yang G., Su F., Hu D., Ruan C., Che P., Zhang Y., Wang J. (2022). Optimization of the Extraction Process and Antioxidant Activity of Polysaccharide Extracted from *Centipeda minima*. Chem. Biodivers..

[9] Rod-in W., Talapphet N., Monmai C., Jang A.Y., You S., Park W.J. (2021). Immune enhancement effects of Korean ginseng berry polysaccharides on RAW264.7 macrophages through MAPK and NF-κB signalling pathways. Food Agric. Immunol..

[10] Zhan Q., Chen Y., Guo Y., Wang Q., Wu H., Zhao L. (2022). Effects of selenylation modification on the antioxidative and immunoregulatory activities of polysaccharides from the pulp of *Rose laevigata* Michx fruit. Int. J. Biol. Macromol..

[11] Kaur R., Shekhar S., Prasad K. (2024). Functional beverages: Recent trends and prospects as potential meal replacers. Food Mater. Res..

[12] Awodire E.F., Ademosun A.O., Ajeigbe O.F., Oboh G. (2023). Functional foods and their applications in managing globally common disease-linked comorbidities. Food Mater. Res..

[13] Wang Z., Wang L., Yu X., Wang X., Zheng Y., Hu X., Zhang P., Sun Q., Wang Q., Li N. (2024). Effect of polysaccharide addition on food physical properties: A review. Food Chem..

[14] Kong D., Zhang M., Mujumdar A.S., Yu D. (2023). New drying technologies for animal/plant origin polysaccharide-based future food processing: Research progress, application prospects and challenges. Food Biosci..

[15] Ali M.Q., Ahmad N., Azhar M.A., Munaim M.S.A., Ruslan N.F. (2025). Fruit and vegetable by-products: Extraction of bioactive compounds and utilization in food biodegradable material and packaging. Food Mater. Res..

[16] Jing Y., Hu Y., Wang Z., Tao C., Zhang S., Hu B., Li Z. (2025). Research progress on extraction, structure, bioactivity, structure-activity relationship and product applications of polysaccharides from Mori fructus. Food Med. Homol..

[17] Zhu Y., He Z., Bao X., Wang M., Yin S., Song L., Peng Q. (2021). Purification, in-depth structure analysis and antioxidant stress activity of a novel pectin-type polysaccharide from *Ziziphus jujuba* cv. *Muzaoresidue*. J. Funct. Foods.

[18] Zhou R., Teng L., Zhu Y., Zhang C., Yang Y., Chen Y. (2021). Preparation of Amomum longiligulare polysaccharides 1- PLGA nanoparticle and its immune enhancement ability on RAW264.7 cells. Int. Immunopharmacol..

[19] Liu X., Yu X., Zhang X., Li F., Zhang X. (2021). Preparation of polysaccharides from *Osmunda japonica* (Thunb) with the potential of food additives: Structural features and functional properties. J. Food Process. Preserv..

[20] Huang D., Li F., Yang A., Wang J., Xie M., Mao M., Li J., Zhang X., Qu Q., Xiong R. (2023). Optimized extraction of polysaccharide from Pinus elliottii: Characterization, antioxidant, and moisture-preserving activities. Fitoterapia.

[21] Cui Y., Li B. (2025). Hypoglycemic effects of edible fungus polysaccharides: A mini review. Food Med. Homol..

[22] Fan W., Fan X., Xie Y., Yan X., Tao M., Zhao S., Yu B., Li R. (2025). Research progress on the anti-aging effects and mechanisms of polysaccharides from Chinese herbal medicine. Food Med. Homol..

[23] Xu Y., Cao H., He J. (2025). Research advances in okra polysaccharides: Green extraction technology, structural features, bioactivity, processing properties and application in foods. Food Res. Int..

[24] Keerthana C.S., Kumari R., Beura M., Sharan S., Sharma S.K., Ganjoo A., Dahuja A., Krishnan V. (2025). Unveiling the glucan profile: A comparative study of Lion’s Mane and Shiitake mushrooms. Nat. Prod. Res..

[25] Li Y., Liu J., Pei D., Di D. (2025). Structural Characterization of, and Protective Effects Against, CoCl_2_-Induced Hypoxia Injury to a Novel Neutral Polysaccharide from *Lycium barbarum* L.. Foods.

[26] Ding H., Zhu X., Liu J., Si J., Wu L. (2025). Plant endophytic fungal polysaccharides and their activities: A review. Int. J. Biol. Macromol..

[27] Liang H., Ma Y., Zhao Y., Qayyum N., He F., Tian J., Sun X., Li B., Wang Y., Wu M. (2025). A Review on the Extraction, Structural Analysis, and Antitumor Mechanisms of *Sanghuangporus* Polysaccharides. Foods.

[28] Zhang S., Chen L., Shang N., Wu K., Liao W. (2025). Recent Advances in the Structure, Extraction, and Biological Activity of *Sargassum fusiforme* Polysaccharides. Mar. Drugs.

[29] Wang T., Zou X., Zhang H., Li J., Peng X., Ju R., Jia Z., Wen Z., Li C. (2025). Ultrasound-Assisted Extraction of Polysaccharides from Mulberry Leaves Using Response Surface Methodology: Purification and Component Identification of Extract. Molecules.

[30] Yang Z.J., Zhang Y.H., Yao X.Q., Gao W.Y., Jin X.H. (2012). Anti-inflammatory Activity of Chemical Constituents Isolated from *Peristrophe roxburghiana*. Lat. Am. J. Pharm..

[31] Aluko E.O., Adejumobi O.A., Fasanmade A.A. (2019). *Peristrophe roxburghiana* leaf extracts exhibited anti-hypertensive and antilipidemic properties in L-NAME hypertensive rats. Life Sci..

[32] Thuy N.M., Tien V.Q., Van Tai N., Minh V.Q. (2022). Effect of Foaming Conditions on Foam Properties and Drying Behavior of Powder from Magenta (*Peristrophe roxburghiana*) Leaves Extracts. Horticulturae.

[33] Huang J., Su M., Wang R., Wu H., Dong F., Li Y., Yao Y. (2021). Research on Dyeing Process of *Peristrophe baphica* (*Spreng*) *Bremek* to Rice. Mod. food.

[34] Hong Z., Lu K., Huang W., Gong J. (2021). The optimization of extraction method of pigment from *Peristrophe roxburghina* and its dyeing on glutinous rice. Food Ferment. Ind..

[35] Mazarei F., Jooyandeh H., Noshad M., Hojjati M. (2017). Polysaccharide of caper (*Capparis spinosa* L.) Leaf: Extraction optimization, antioxidant potential and antimicrobial activity. Int. J. Biol. Macromol..

[36] Yu L., Zhang H., Yang L., Tian K. (2019). Optimization of purification conditions for papain in a polyethylene glycol-phosphate aqueous two-phase system using quaternary ammonium ionic liquids as adjuvants by BBD-RSM. Protein Expr. Purif..

[37] Zhao D., Zhou X., Gaong X., Quan W., Gao G., Zhao C. (2024). Optimization of ultrasound-assisted extraction of flavonoids from *Emilia prenanthoidea* DC. using response surface methodology and exploration of the ecological factors on total flavonoid and antioxdant activity. Food Med. Homol..

[38] Nurmamat E., Xiao H.X., Zhang Y., Jiao Z.W. (2018). Effects of Different Temperatures on the Chemical Structure and Antitumor Activities of Polysaccharides from *Cordyceps militaris*. Polymers.

[39] Yang D., Lin F., Huang Y., Ye J., Xiao M. (2020). Separation, purification, structural analysis and immune-enhancing activity of sulfated polysaccharide isolated from sea cucumber viscera. Int. J. Biol. Macromol..

[40] Kumar V., Agrawal D., Bommareddy R.R., Islam M.A., Jacob S., Balan V., Singh V., Thakur V.K., Navani N.K., Scrutton N.S. (2024). Arabinose as an overlooked sugar for microbial bioproduction of chemical building blocks. Crit. Rev. Biotechnol..

[41] Jankute M., Grover S., Rana A.K., Besra G.S. (2011). Arabinogalactan and Lipoarabinomannan Biosynthesis: Structure, Biogenesis and Their Potential as Drug Targets. Future Microbiol..

[42] Shimoda R., Okabe K., Kotake T., Matsuoka K., Koyama T., Tryfona T., Liang H.-C., Dupree P., Tsumuraya Y. (2014). Enzymatic fragmentation of carbohydrate moieties of radish arabinogalactan-protein and elucidation of the structures. Biosci. Biotech. Biochem..

[43] Uemura Y., Asakuma S., Nakamura T., Arai I., Taki M., Urashima T. (2005). Occurrence of a unique sialyl tetrasaccharide in colostrum of a bottlenose dolphin (*Tursiops truncatus*). BBA Gen. Subj..

[44] Yasukochi T., Fukase K., Suda Y., Takagaki K., Endo M., Kusumoto S. (1997). Enzymatic synthesis of 4-methylumbelliferyl glycosides of trisaccharide and core tetrasaccharide, Gal(β1-3)Gal(β1-4)Xyl and GlcA(β1-3)Gal(β1-3)Gal(β1-4)Xyl, corresponding to the linkage region of proteoglycans. Bull. Chem. Soc. Jpn..

[45] Hakkarainen B., Kenne L., Lahmann M., Oscarson S., Sandström C. (2007). NMR study of hydroxy protons of di- and trimannosides, substructures of Man-9. Magn. Reson. Chem..

[46] Royer P.J., Emara M., Yang C.X., Al-Ghouleh A., Tighe P., Jones N., Sewell H.F., Shakib F., Martinez-Pomares L., Ghaemmaghami A.M. (2010). The Mannose Receptor Mediates the Uptake of Diverse Native Allergens by Dendritic Cells and Determines Allergen-Induced T Cell Polarization through Modulation of IDO Activity. J. Immunol..

[47] Witkowski A., Carta S., Lu R., Yokoyama S., Rubartelli A., Cavigiolio G. (2019). Oxidation of methionine residues in human apolipoprotein A-I generates a potent pro-inflammatory molecule. J. Biol. Chem..

[48] Yao Y.F., Wang L.F., Chen S.M., Wu R.T., Long F.Y., Li W.J. (2022). Antinociceptive and anti-inflammatory activities of ethanol-soluble acidic component from *Ganoderma atrum* by suppressing mannose receptor. J. Funct. Foods.

[49] Romdhane M.B., Haddar A., Ghazala I., Jeddou K.B., Helbert C.B., Ellouz-Chaabouni S. (2017). Optimization of polysaccharides extraction from watermelon rinds: Structure, functional and biological activities. Food Chem..

[50] Peng Y., Ma F., Hu L., Deng Y., He W., Tang B. (2022). Strontium based Astragalus polysaccharides promote osteoblasts differentiation and mineralization. Int. J. Biol. Macromol..

[51] Zhao Z., Yuwen W., Duan Z., Zhu C., Fan D. (2024). Novel Collagen Analogs with Multicopy Mucin-Type Sequences for Multifunctional Enhancement Properties Using SUMO Fusion Tags. J. Agric. Food Chem..

[52] Ellerbrock R.H., Gerke H.H. (2021). FTIR spectral band shifts explained by OM–cation interactions. J. Plant Nutr. Soil Sci..

[53] Fang C.L., Huang J.R., Pu H.Y., Yang Q., Chen Z.G., Zhu Z.B. (2021). Cold-water solubility, oil-adsorption and enzymolysis properties of amorphous granular starches. Food Hydrocoll..

[54] Anvari M., Tabarsa M., Cao R., You S., Joyner H.S., Behnam S., Rezaei M. (2016). Compositional characterization and rheological properties of an anionic gum from *Alyssum homolocarpum* seeds. Food Hydrocoll..

[55] Xu Y.Q., Liu N.Y., Fu X.T., Wang L.X., Yang Y., Ren Y.Y., Liu J.Y., Wang L.B. (2019). Structural characteristics, biological, rheological and thermal properties of the polysaccharide and the degraded polysaccharide from raspberry fruits. Int. J. Biol. Macromol..

[56] Iijima M., Hatakeyama T., Hatakeyama H. (2021). DSC and TMA Studies of Polysaccharide Physical Hydrogels. Anal. Sci..

[57] Ilyasov I.R., Beloborodov V.L., Selivanova I.A., Terekhov R.P. (2020). ABTS/PP Decolorization Assay of Antioxidant Capacity Reaction Pathways. Int. J. Mol. Sci..

[58] Dumandan N.G., Acda R.D.P., Kagaoan A.C.T., Tumambing C.R. (2024). Nutraceutical prospects of phenolic compounds from desugared sugarcane extract: An in vitro study of its bioaccessibility and bioactivity. Food Mater. Res..

[59] Xu J., Tian T., Zhao Y. (2025). Effect of extrusion processing on physicochemical and functional properties of water-soluble dietary fiber and water-insoluble dietary fiber of whole grain highland barley. Food Med. Homol..

[60] Chen J., Li G., Chen X., Liao L., He Y., Ye F., Chen G. (2025). Nutritional value and antioxidant activity of *Artemisia princeps*, an edible plant frequently used in folk food in the Xiangxi region. Food Med. Homol..

[61] Ziyatdinova G., Budnikov H. (2021). Analytical Capabilities of Coulometric Sensor Systems in the Antioxidants Analysis. Chemosensors.

[62] Gulcin I., Alwasel S.H. (2023). Fe^3+^ Reducing Power as the Most Common Assay for Understanding the Biological Functions of Antioxidants. Processes.

[63] Lushchak V.I. (2014). Free radicals, reactive oxygen species, oxidative stress and its classification. Chem. Biol. Interact..

[64] López-Alarcón C., Fuentes-Lemus E., Figueroa J.D., Dorta E., Schöneich C., Davies M.J. (2020). Azocompounds as generators of defined radical species: Contributions and challenges for free radical research. Free Radic. Biol. Med..

[65] Zhan Q., Wang Q., Liu Q., Guo Y., Gong F., Hao L., Wu H., Dong Z. (2021). The antioxidant activity of protein fractions from Sacha inchi seeds after a simulated gastrointestinal digestion. LWT.

[66] Zhang Z., Wei T., Hou J., Li G., Yu S., Xin W. (2002). Reactive oxygen species are involved in lysophosphatidic acid-induced apoptosis in rat cerebellar granule cells. Res. Chem. Intermed..

[67] Zhou D., Gang D., Furao L., Hui W., Qiping Z. (2021). Purification and comparative study of bioactivities of a natural selenized polysaccharide from *Ganoderma lucidum* mycelia. Int. J. Biol. Macromol..

[68] Yadav A., Singh A., Rawat P., Pal A., Yadav P., Tyagi V., Singh S.K., Sethi A., Singh R.P. (2024). Investigation of corticosteroid-NSAID prodrugs for TNF-α and IL6 receptors, their characterization, single crystal XRD and DFT studies: A combined experimental and theoretical approach. J. Mol. Struct..

[69] Zhan Q.P., Wang Q., Lin R.G., He P., Lai F.R., Zhang M.M., Wu H. (2020). Structural characterization and immunomodulatory activity of a novel acid polysaccharide isolated from the pulp of Michx fruit. Int. J. Biol. Macromol..

[70] Wang F., Liu L., Zhu Z., Aisa H.A., Xin X. (2024). Anti-inflammatory effect and mechanism of active parts of Artemisia mongolica in LPS-induced Raw264.7 cells based on network pharmacology analysis. J. Ethnopharmacol..

