# Supplementary material for: Immune Enhancement Effects and Extraction Optimization of Polysaccharides from Peristrophe roxburghiana
Source: Antioxidants (Basel). 2025 Sep 1;14(9):1072. doi: 10.3390/antiox14091072 (PMC12466801; doi:10.3390/antiox14091072)
Supplement: Supplementary file 1 [file antioxidants-14-01072-s001.zip › antioxidants-3823574-supplementary.pdf]

Supplementary Materials

Immune enhancement effects and extraction optimization of polysaccharides from *Peristrophe roxburghiana*

Table S1. Information on the standard substances of this project.

Name	Abbreviation	CAS	Molecular Formula
Fucose	Fuc	2438-80-4	$C_6H_{12}O_5$
Rhamnose	Rha	10030-85-0	$C_6H_{12}O_5$
Arabinose	Ara	5328-37-0	$C_5H_{10}O_5$
Galactose	Gal	26566-61-0	$C_6H_{12}O_6$
Glucose	Glc	50-99-7	$C_6H_{12}O_6$
Xylose	Xyl	58-86-6	$C_5H_{10}O_5$
Mannose	Man	3458-28-4	$C_6H_{12}O_6$
Fructose	Fru	57-48-7	$C_6H_{12}O_6$
Ribose	Rib	50-69-1	$C_5H_{10}O_5$
Galacturonic Acid	Gal-UA	14982-50-4	$C_6H_{10}O_7$
Glucuronic Acid	Glc-UA	6556-12-3	$C_6H_{10}O_7$
Mannuronic Acid	Man-UA	6814-36-4	$C_6H_{10}O_7$
Guluronic Acid	Gul-UA	15769-56-9	$C_6H_{10}O_7$

Table S2. Information on the standard substances of this project.

Compound	LOD	LOQ	Intraday precision (RSD%)	Interday precision (RSD%)
Fuc	0.1ug/ml	0.4ug/ml	0.65	0.93
Rha	0.2ug/ml	0.4ug/ml	0.12	1.14
Ara	0.1ug/ml	0.4ug/ml	0.72	1.01
Gal	0.1ug/ml	0.4ug/ml	0.24	2.23
Glc	0.2ug/ml	0.4ug/ml	0.40	1.98
Xyl	0.2ug/ml	0.4ug/ml	0.25	0.80
Man	0.2ug/ml	0.5ug/ml	0.69	1.13
Fru	0.5ug/ml	0.6ug/ml	0.94	1.84
Rib	0.2ug/ml	0.6ug/ml	1.04	0.98
Gal-UA	0.5ug/ml	0.6ug/ml	0.82	1.55
Gul-UA	0.5ug/ml	0.6ug/ml	0.59	1.39
Glc-UA	0.5ug/ml	0.6ug/ml	0.88	2.68
Man-UA	0.5ug/ml	0.6ug/ml	0.54	1.65

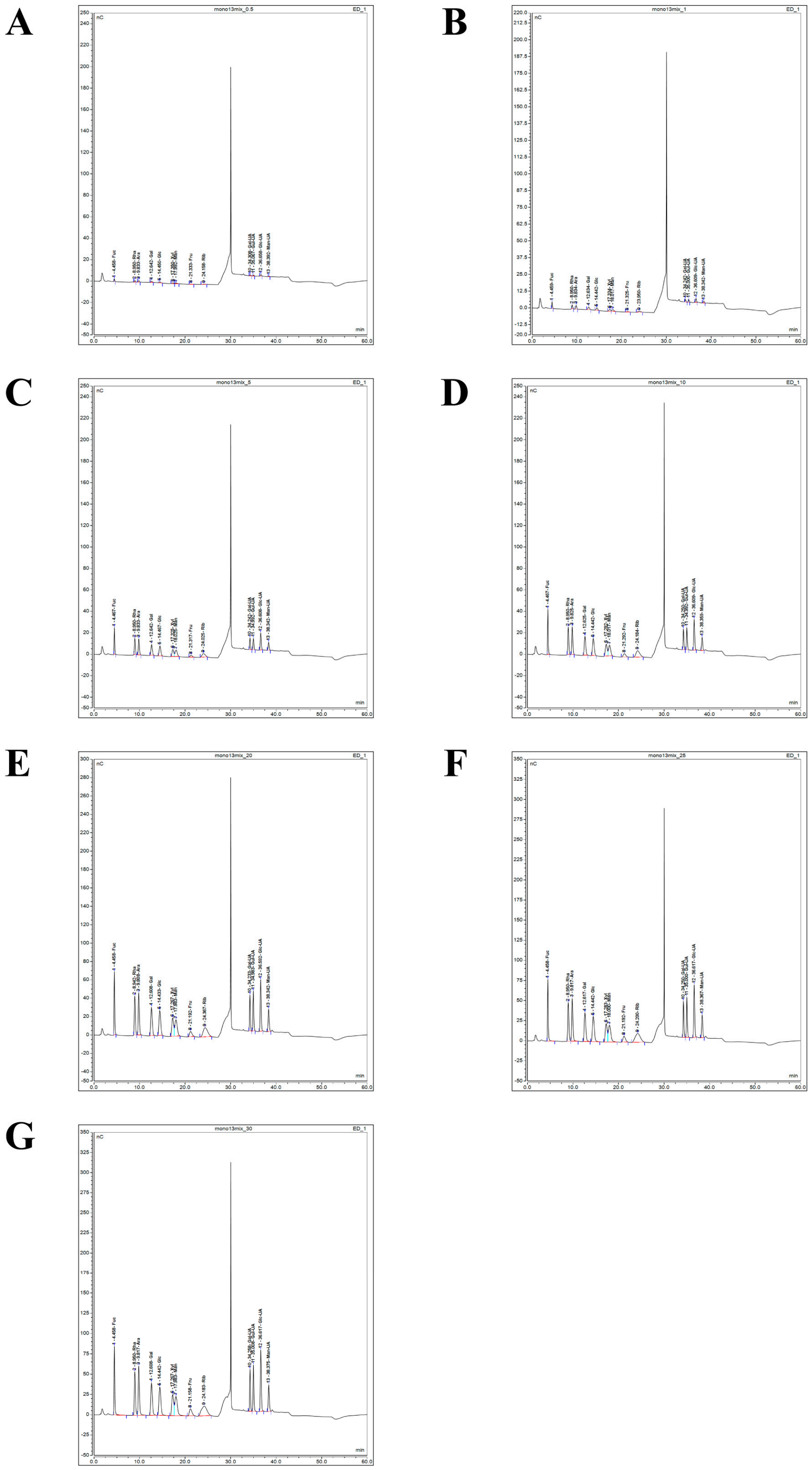

Figure S1. The liquid chromatograms of the monosaccharide standard samples.

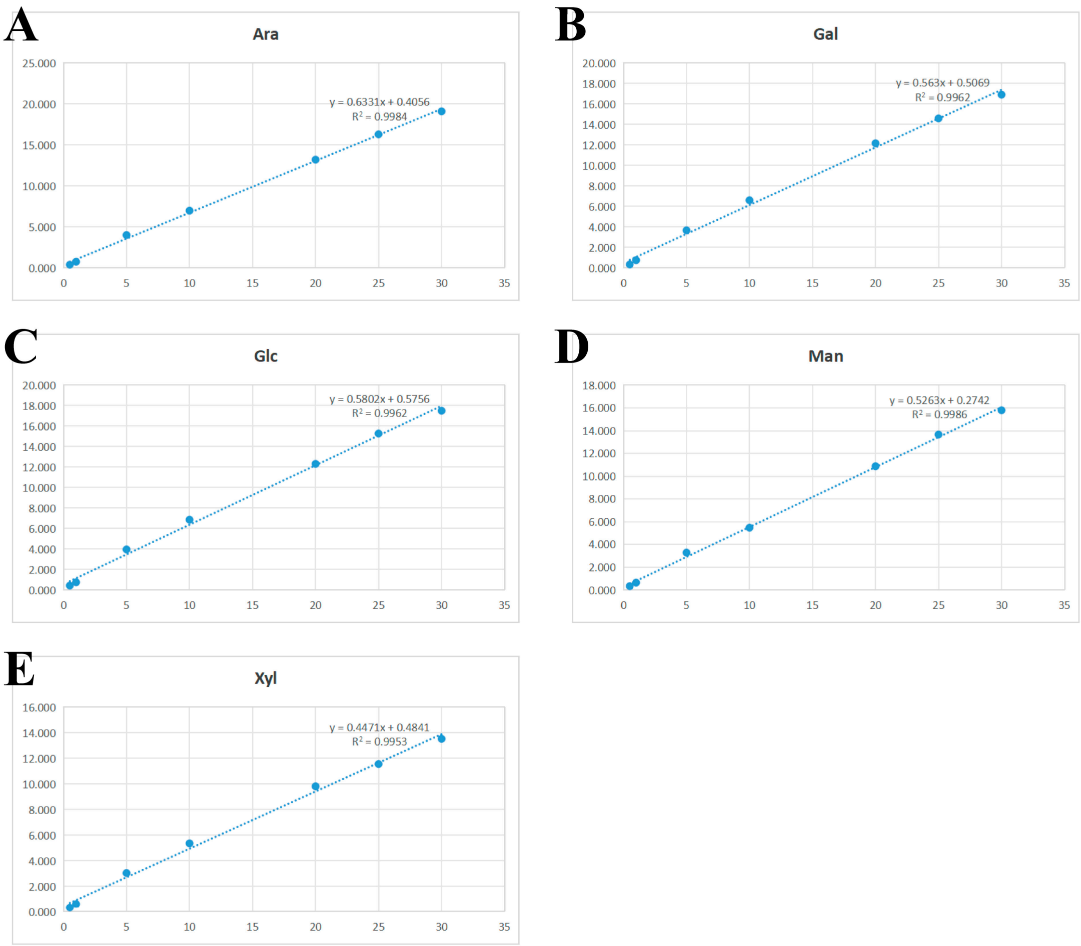

Figure S2. Standard curves for each monosaccharide (Ara, Gal, Glc, Man, and Xyl). The abscissa represents the mass concentration (μg/ml), and the ordinate represents the peak area of the liquid chromatography (nC*min).

Table S3. Monosaccharide composition of CPPRs.

Name	Project	Fuc	Ara	Rha	Gal	Glc	Xyl	Man	Fru	Rib	Gal-UA	Gul-UA	Glc-UA	Man-UA	Total content of each component
CPPRs	Mass ratio (μg/mg)	0	100.7271	0	54.588	43.1004	14.1147	11.6107	0	0	0	0	0	0	224.1409
	Molar mass ratio (%)	0.00%	48.92%	0.00%	22.09%	17.44%	6.85%	4.70%	0.00%	0.00%	0.00%	0.00%	0.00%	0.00%	100%
